# Age-adjusted Charlson Comorbidity Index as effective predictor for in-hospital mortality of patients with cardiac arrest: a retrospective study

**DOI:** 10.1186/s12873-022-00769-4

**Published:** 2023-01-26

**Authors:** Nan Zhang, Qingting Lin, Hui Jiang, Huadong Zhu

**Affiliations:** Emergency Department, State Key Laboratory of Complex Severe and Rare Diseases, Peking Union Medical College Hospital, Chinese Academy of Medical Science and Peking Union Medical College, Beijing, 100730 China

**Keywords:** Cardiac arrest, Age-adjusted Charlson Comorbidity Index, In-hospital mortality, Length of hospital stay

## Abstract

**Background:**

Cardiac arrest is currently one of the leading causes of mortality in clinical practice, and the Charlson Comorbidity Index (CCI) is widely utilized to assess the severity of comorbidities. We aimed to evaluate the relationship between the age-adjusted CCI score and in-hospital mortality in intensive care unit (ICU) patients with the diagnosis of cardiac arrest, which is important but less explored previously.

**Methods:**

This was a retrospective study including patients aged over 18 years from the MIMIC-IV database. We calculated the age-adjusted CCI using age information and ICD codes. The univariate analysis for varied predictors’ differences between the survival and the non-survival groups was performed. In addition, a multiple factor analysis was conducted based on logistic regression analysis with the primary result set as hospitalization death. An additional multivariate regression analysis was conducted to estimate the influence of hospital and ICU stay.

**Results:**

A total of 1772 patients were included in our study, with median age of 66, among which 705 (39.8%) were female. Amongst these patients, 963 (54.3%) died during the hospitalization period. Patients with higher age-adjusted CCI scores had a higher likelihood of dying during hospitalization (*P* < 0.001; OR: 1.109; 95% CI: 1.068–1.151). With the age-adjusted CCI incorporated into the predictive model, the area under the receiver operating characteristic curve was 0.794 (CI: 0.773–0.814), showing that the prediction model is effective. Additionally, patients with higher age-adjusted CCI scores stayed longer in the hospital (*P* = 0.026, 95% CI: 0.056–0.896), but there was no significant difference between patients with varied age-adjusted CCI scores on the days of ICU stay.

**Conclusion:**

The age-adjusted CCI is a valid indicator to predict death in ICU patients with cardiac arrest, which can offer enlightenment for both theory literatures and clinical practice.

## Background

The incidence of and mortality from cardiac arrest are still very high around the world, and it is one of the main causes of death [1]. A recent review noted that comorbidities are important confounders that may affect the outcomes, including mortality, in observational studies [2]. Adjustment for comorbidity is usually neglected in cardiac arrest research, for both in-hospital cardiac arrest (IHCA) and out-of-hospital cardiac arrest (OHCA) [3], which is the focus in this article.

The Charlson Comorbidity Index (CCI), a standardized score calculated as just a simple weighted sum of comorbidity item scores, was developed in 1987 by Mary E. Charlson, and has been considered the gold-standard tool in clinical research as a prognostic index to predict mortality [4, 5]. The original version of the CCI was based on 19 items corresponding to different clinical comorbidities [4]. Subsequently, different versions of the CCI have been developed based on different sources of data, including the age-adjusted CCI, ICD-9 code based CCI [6–8] and ICD-10 code based CCI [9, 10].

Many previous studies have demonstrated that the CCI score has a close association with mortality [5]. The combined age-comorbidity score was also proven to be a valid predictor of clinical outcomes in patients with different diseases [11–13]. However, the relationship between comorbidity and survival in patients with cardiac arrest remains uncertain. Some studies have shown that comorbidities have a significant predictive power for clinical outcomes in patients with cardiac arrest [14–17] but other studies failed to find similar results [18, 19].

Considering that age is one of the important influencers related to the prognosis of patients with cardiac arrest [20, 21], we evaluated the relationship between mortality in ICU patients with cardiac arrest and their age-adjusted CCI score. Through the systematical retrospective study, we expect to offering some enlightenments for both the theoretical literature and clinical practice.

## Methods

This was a retrospective study using data from the MIMIC-IV database [22], a public database developed and maintained by the Laboratory for Computational Physiology at Massachusetts Institute of Technology [23]. The MIMIC database is a large, open and single-center database containing information on patients admitted to critical care units at a large tertiary care hospital [23]. We used SQL and STATA 17.0 to extract and merge the data.

Patients entering the ICU whose age was over 18 with a diagnosis of cardiac arrest (with ICD codes of ’42754’ for the records meeting ICD version 9, ’I46’, ’I462’, ’I468’, and ’I469’ for the records meeting ICD version 10) were included. Sample patients were divided into two groups according to whether they experienced hospital mortality. The age-adjusted CCI was calculated based on the patients’ age and ICD codes.

Among other factors entering the analysis, the vital signs like blood pressure are easy to obtain, and previous studies have suggested that abnormal vital signs measured routinely are associated with poor prognosis of patients with cardiac arrest [24, 25], which should be included into the model. Previous studies have also shown that the level of lactic acid at admission is closely related to the increase of in-hospital mortality in ICU patients [26], which we also included. Besides, Sequential Organ Failure Assessment (SOFA) is a scoring system to assess the severity of multiple organ dysfunction in ICU patients [27], and the Glasgow Coma Scale (GCS) is widely used for comatose patients in intensive care [28]. These two scores are easy to operate and obtain, which also enter our analysis. In other aspects, previous studies have suggested that age, sex, as well as other characteristics are lactated with prognosis of patients with cardiac arrest [14, 20], and should be considered as well.

The data used in our study are from a public database, so individual patient consent was not needed. The authors acquired permission to use the database by passing an online exam and following the relevant guidelines during the research. The MIMIC-IV database has received ethical approval from the Institutional Review Boards (IRBs) at Beth Israel Deaconess Medical Center (BIDMC) and MIT. And the data used in our study were obtained from a public database, which do not contain protected health information, so individual patient consent was not needed. The authors obtained permission to use the data.

All procedures were followed in accordance with the Helsinki Declaration of 1975.

### Outcomes

The primary outcome was in-hospital death. The secondary outcomes were the ICU and hospitalization stays of the surviving patients.

### Statistical analysis

The patients’ characteristics were subjected to descriptive statistical analysis. Categorical variables are presented as frequencies and percentages. Continuous variables are presented as the mean value with standard deviation (SD) in parentheses if the variables conformed to a normal distribution. Otherwise, they are presented using the median (interquartile range [IQR]). The majoring vital signs are recoded during the first 24 h after the ICU admission. Shapiro‒Wilk test was used to test whether the continuous variables conformed to a normal distribution. Categorical variables were compared between the survivors and the non-survivors using the Pearson chi-squared test or Fisher’s exact test. We used the Mann‒Whitney U test for variables with a nonnormal distribution and Student’s t-test for data with a normal distribution.

Then, a multivariable logistic regression analysis was conducted to test whether age-adjusted CCI was associated with in-hospital death. We also included into the model other important factors presented relative to the in-hospital death from previous studies. The dependent variable was set as the outcome of whether the patients died in hospital or not. The independent variables were set as the variables with significant differences (*P* value < 0.1) between the survivors and the non-survivors, which can guarantee that the model built is effective and efficient with the most important influencers included. We also tried other methods like step-wise regression to decide the factors to enter the prediction model and find the univariate regression method the most effective and efficient. We chose to report the odds ratio (OR) with 95% confidence intervals (95% CI). Based on the results from the logistic regression analysis, the area under the ROC curve was plotted to exhibit the predictive accuracy of the models. ROC curve is usually used to evaluate the discrimination of the models. Simultaneously, we created a nomogram with the factors that had significant impacts on in-hospital mortality according to the multiple logistic regression.

Finally, we performed a multiple linear regression analysis to evaluate the factors influencing the length of stay for both hospitalization and ICU admission. This analysis focused on the patients who survived. Due to some missing variables, multiple imputation methods were applied to improve the quality of the dataset. To be specific, Predictive Mean Matching imputation is chosen, where the interpolation value is a combination of the predicted value of the regression model and a random error term.

All statistical analyses were performed with STATA (version 17.0) and R (version 13.0) software. A two-sided *p* value less than 0.05 means a significant result if not specifically stated otherwise.

## Results

### Study population

**Fig. 1:**
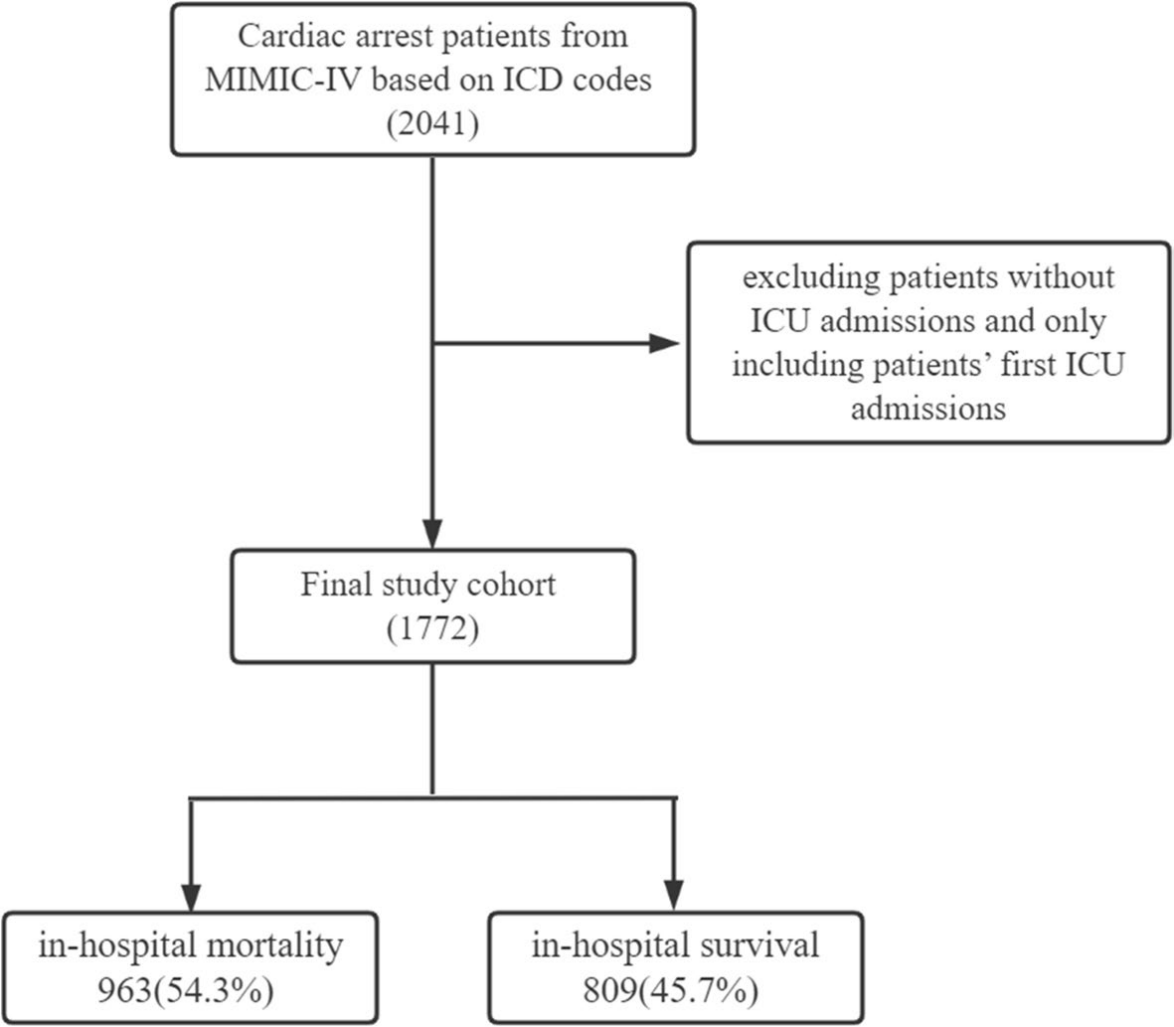
The inclusion and exclusion criteria to select the final study cohort

Based on the ICD codes, a total of 2041 adult patients were diagnosed with cardiac arrest in MIMIC-IV. Among these 2041 original candidate patients, those who were recorded as their first admission to the ICU were included in our subsequent analysis, 1772 eligible patients (shown in Fig. 1).

### Characteristics of the study patients

All of the patients’ baseline characteristics are summarized in Table 1. Overall, their average age was 66 (55,78) years and there were 705 (39.8%) female patients. Among these patients, 963 (54.3%) (553 men and 410 women) died during hospitalization, with an in-hospital mortality rate of 54.3%.

**Table 1:** Patient characteristics

	All patients	Hospital mortality		
	(*n* = 1,772)	Yes (*n* = 963)	No (*n* = 809)	*P*-value
**Age (years)**	66(55,78)	69(55,79)	64(54,75)	< 0.001
**Female sex (n, %)**	705(39.8)	410(42.6)	295(36.5)	0.009
ICD-I462 **(n, %)**	151(8.5)	43(4.5)	108(13.3)	< 0.001
**BMI (kg/m**^**2**^**)(IQR)**	27.4(23.7,32.0)	26.8(23.1,31.9)	27.9(24.3,32.3)	< 0.001
**Admission location (n, %)**	955(53.9)	537(55.8)	418(51.7)	0.085
**SBP (min, mmHg) (IQR)**	83(71,95)	79(63,91)	86(77,98)	< 0.001
**SBP (max, mmHg) (IQR)**	147(132,166)	146(129,165)	149(135,167)	0.001
**DBP (min, mmHg) (IQR)**	43(34,51)	40(31,49)	45(38,53)	< 0.001
**DBP (max, mmHg) (IQR)**	87(74,102)	86(73,103)	88(77,100)	0.246
** h (min, bpm) (IQR)**	66(55,79)	68(56,82)	63(55,75)	< 0.001
** h (max, bpm) (IQR)**	104(89,124)	109(93,128)	99(86,117)	< 0.001
**RR (min, bpm) (IQR)**	13(10,16)	13(10,16)	12(10,15)	< 0.001
**RR (max, bpm) (IQR)**	28(24,32)	29(25,34)	27(24,31)	< 0.001
**T (min, ℃) (IQR)**	36.3(35.3,36.6)	36.0(34.7,36.5)	36.4(35.8,36.7)	< 0.001
**T (max, ℃) (IQR)**	37.1(36.6,37.7)	37.0(36.2,37.6)	37.2(36.9,37.7)	< 0.001
**SpO**_**2**_**(min, %) (IQR)**	92(87,95)	91.(82,95)	93(90,95)	< 0.001
**SpO**_**2**_**(max, %) (IQR)**	100(100,100)	100(100,100)	100(100,100)	< 0.001
**GCS (min) (IQR)**	14(7,15)	14(5,15)	14(9,15)	0.758
**Lac (min, mmol/L) (IQR)**	1.9(1.3,3.4)	2.5(1.5,4.6)	1.6(1.1,2.3)	< 0.001
**Lac (max, mmol/L) (IQR)**	14(7,15)	4.8(2.3,8.5)	2.8(1.7,5)	< 0.001
**Sofa (IQR)**	9(5,12)	10(6,13)	7(4,11)	< 0.001
Respiration **(IQR)**	2(0,4)	2(0,4)	2(0,4)	< 0.001
Coagulation **(IQR)**	0(0,1)	0(0,1)	0(0,1)	< 0.001
Liver **(IQR)**	0(0,0)	0(0,1)	0(0,0)	< 0.001
Cardiovascular **(IQR)**	3(1,4)	4(1,4)	1(1,4)	< 0.001
CNS **(IQR)**	1(0,3)	1(0,4)	1(0,3)	0.600
Renal **(IQR)**	1(0,3)	2(1,3)	1(0,2)	< 0.001
**Age-adjusted CCI (IQR)**	6(4,8)	6(4,9)	6(4,8)	< 0.001
Age-Score **(IQR)**	3(2,4)	4(2,4)	3(2,4)	< 0.001
Myocardial infarct (n, %)	517(29.2)	262(27.2)	255(31.5)	0.047
Congestive heart failure (n, %)	720(40.6)	355(36.9)	365(45.1)	< 0.001
Peripheral vascular disease (n, %)	259(14.6)	152(15.8)	107(13.2)	0.129
Cerebrovascular disease (n, %)	282(15.9)	176(18.3)	105(13)	0.002
Dementia (n, %)	59(3.3)	35(3.6)	24(3.0)	0.435
Chronic pulmonary disease (n, %)	454(25.6)	246(25.5)	208(25.7)	0.937
Rheumatic disease (n, %)	58(3.3)	35(3.6)	23(2.8)	0.351
Peptic ulcer disease (n, %)	52(2.9)	24(2.5)	28(3.5)	0.229
Mild liver disease (n, %)	280(15.8)	172(17.9)	108(13.3)	0.010
Diabetes without complications (n, %)	478(27.0)	271(28.1)	208(25.7)	0.251
Diabetes with complications (n, %)	245(13.8)	115(11.9)	129(15.9)	0.015
Paraplegia (n, %)	65(3.7)	35(3.6)	30(3.7)	0.934
Renal disease (n, %)	517(29.2)	279(29)	239(29.5)	0.792
Malignant cancer (n, %)	200(11.3)	137(14.2)	63(7.8)	< 0.001
Severe liver disease (n, %)	83(4.7)	61(6.3)	22(2.7)	< 0.001
Metastatic solid tumor (n, %)	83(4.7)	69(7.2)	14(1.7)	< 0.001
AIDS (n, %)	10(0.6)	7(0.7)	3(0.4)	0.319
**Sepsis (n, %)**	1179(66.5)	649(67.4)	530(65.5)	0.403
**Defibrillation (n, %)**	87(4.9)	41(4.3)	46(5.7)	0.166
**MV (n, %)**	1189(67.1)	682(70.8)	507(62.7)	< 0.001

IQR is reported for continuous variable

*ICD-I462* whether the patient is with cardiac arrest due to underlying cardiac condition, *BMI* Body mass index, *SBP* Systolic blood pressure, *DBP* Diastolic blood pressure, *HR* Heart rate, *RR* Respiratory rate, *T* Temperature, *SpO2* pulse oxygen saturation, *GCS* Glasgow Coma Scale, *Lac* Lactate, *AIDS* Acquired Immune Deficiency Syndrome, *MV* Mechanical ventilation

The patients were then divided into two groups according to their in-hospital death outcomes. Patients who died in the hospital had lower blood pressure and GCS. The heart rates, respiratory rates and lactate levels among those who experienced hospitalization death were higher than those of patients who survived in the hospital. The use of ventilation was also more common in the death group. However, there was no significant difference in sepsis or defibrillation between the two groups.

There were differences between the two groups for total SOFA and CCI scores. There were differences in almost all subcomponents of the SOFA scores between the two groups. Among the CCI components, there were differences in several comorbidities, including myocardial infarct, congestive heart failure, cerebrovascular disease, diabetes with complications, mild or severe liver disease, and cancer with or without metastasis.

### Multivariate logistic regression analysis

**Fig. 2:**
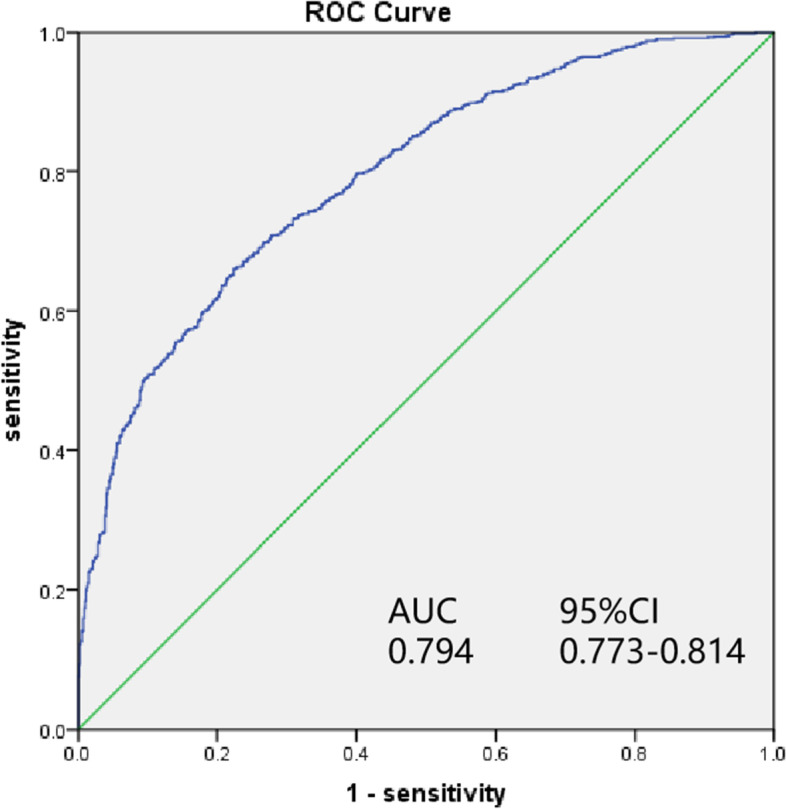
The ROC curve for logistic regression analysis

The logistic regression analysis is presented in Table 2. The results indicated that the age-adjusted CCI score was related to death during hospitalization. The patients with higher age-adjusted CCI scores had a higher probability of dying during hospitalization (*P* < 0.001, OR: 1.109; 95% CI: 1.068–1.151). Other potential risk factors for death included a diagnosis of cardiac arrest, BMI, vital signs, the minimum value of lactate within 24 h of ICU admission, and the use of mechanical ventilation (shown in Table 2). The AUC of the age-adjusted CCI score to predict in-hospital death was 0.794 (shown in Fig. 2).

**Table 2:** The results of logistic regression analysis variables with *p* value less than 0.1

Variables	Primary outcome (In-hospital Death)	
	OR	95% CI
ICD-I462	0.330	0.213–0.509
BMI(kg/m^2^)	0.984	0.969–0.998
DBP (min, mmHg)	0.983	0.974–0.992
h (min, bpm)	1.016	1.009–1.023
h (max, bpm)	0.005	1.007–1.002
T (min,℃)	0.842	0.755–0.939
T (max,℃)	0.861	0.789–0.940
SpO2 (min, %)	0.968	0.955–0.981
SpO2 (max, %)	0.829	0.739–0.930
Lac (min, mmol/L)	1.356	1.255–1.465
Age-adjusted CCI	1.109	1.068–1.151
MV	1.763	1.380–2.252

*ICD-I462* whether the patient is with cardiac arrest due to underlying cardiac condition, *BMI* Body mass index, *DBP* Diastolic blood pressure, *HR* Heart rate, *T* Temperature, *SpO2* pulse oxygen saturation, *GCS* Glasgow Coma Scale, *Lac* Lactate, *AIDS* Acquired; Immune Deficiency Syndrome, *CCI* Charlson comorbidity index, *MV* Mechanical ventilation, *95%CI* 95% confident interval

**Fig. 3:**
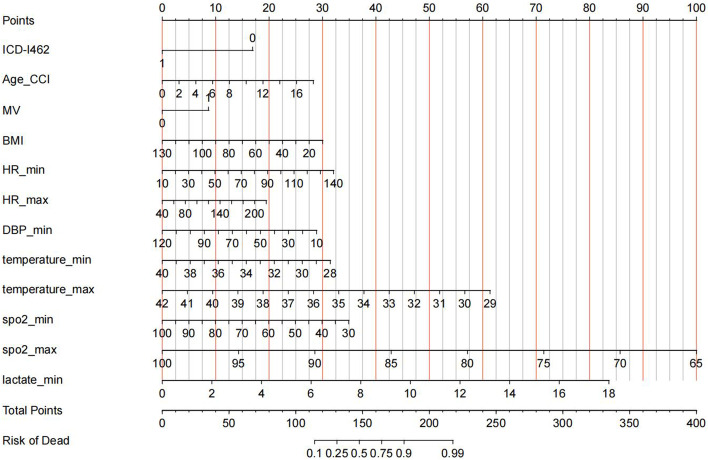
The nomogram to predict the death risk of patients with cardiac arrest ICD-I462: whether the patient is with cardiac arrest due to underlying cardiac condition; BMI: body mass index; DBP: diastolic blood pressure; HR: heart rate; T: Temperature; SpO2: pulse oxygen saturation; GCS: Glasgow Coma Scale; Lac: lactate; AIDS: Acquired Immune Deficiency Syndrome; CCI: Charlson comorbidity index; MV: mechanical ventilation

Additionally, we created a nomogram to predict the death risk of patients, as presented in Fig. 3. The top axis in Fig. 3 plots the scores of the death risk level. The value of each variable that had a significant impact on in-hospital death is given a score on the corresponding point scale axis. Summing up the single scores for those variables, we were able to obtain the total score for the individual patient. Finally, by projecting the total score on the lower total point scale axis, we could estimate the probability of in-hospital death.

### The secondary outcomes

We evaluated some potential factors that might affect the length of stay in the hospital or ICU among the surviving patients. Based on the results of the linear regression model shown in Table 3, patients complicated with sepsis and with lower GCS stay longer in both the hospital and ICU. A higher SOFA or age-adjusted CCI score was associated with a longer stay in the hospital (*P* = 0.026, 96% CI: 0.056–0.896) but not in the ICU.

**Table 3:** The results of multiple regression analysis for survival patients Days in ICU or hospital

Variables	ICU stay		hospital stay	
	Coefficient	95%CI	Coefficient	95%CI
**Female**	0.300	-0.872-1.471	0.221	-2.205-2.647
ICD-I462	-1.264	-2.972-0.445	-2.374	-5.913-1.166
**BMI**	0.050	-0.020-0.121	0.099	-0.047-0.244
**Admission**	-0.006	-1.149-1.137	-2.375	-4.742–0.007
** h(min)**	-0.003	-0.043-0.038	0.004	-0.080-0.088
** h(max)**	0.007	-0.022-0.035	0.065	0.006–0.124
**SBP(min)**	-0.014	-0.059-0.031	0.025	-0.069-0.118
**SBP(max)**	0.009	-0.018-0.036	0.038	-0.019-0.094
**DBP(min)**	0.032	-0.029-0.093	-0.004	-0.131-0.123
**DBP(max)**	-0.011	-0.047-0.024	-0.006	-0.079-0.094
**RR(min)**	-0.040	-0.177-0.096	-0.146	-0.428-0.137
**RR(max)**	0.097	-0.003-0.196	0.163	-0.044-0.369
**T(min)**	-0.827	-1.421–0.233	-0.583	-1.813-0.647
**T(max)**	0.497	-0.165-1.158	-0.033	-1.403-1.337
**SpO2(min)**	-0.020	-0.111-0.071	-0.006	-0.195-0.182
**SpO2(max)**	-0.967	-1.669–0.265	-0.049	-1.503-1.406
**Lac(min)**	-0.036	-0.513-0.440	-0.026	-1.013-0.960
**Lac(max)**	0.102	-0.139-0.343	-0.110	-0.610-0.389
**GCS(min)**	-0.887	-1.059–0.715	-0.711	-1.067–0.355
**SOFA**	-0.019	-0.227-0.189	0.450	0.019–0.880
**Age-adjusted CCI**	0.158	-0.045-0.361	0.476	0.056–0.896
**Sepsis**	2.868	1.449–4.287	3.441	0.501–6.381
**MV**	0.555	-0.872-1.983	-0.905	-3.863-2.053
**Defibrillation**	4.360	1.886–6.834	-0.745	-5.869-4.380

*ICD-I462* whether the patient is with cardiac arrest due to underlying cardiac condition, *BMI* Body mass index, *SBP* Systolic blood pressure, *DBP* Diastolic blood pressure, *HR* Heart rate, *RR* Respiratory rate, *T* Temperature, *SpO2* pulse oxygen saturation, *GCS* Glasgow Coma Scale, *Lac* Lactate, *AIDS* Acquired Immune Deficiency Syndrome, *MV* Mechanical ventilation, *95%CI* 95% confident interval

## Discussion

The prognosis is poor for patients suffering from cardiac arrest around the world. In our retrospective study, the main finding was that increasing age-adjusted CCI was associated with higher in-hospital mortality. Other relevant risk factors included vital signs and minimum value of lactate within 24 h of ICU admission, diagnosis of cardiac arrest, level of BMI, and use of mechanical ventilation.

Comorbidities usually refer to typical chronic diseases that have a significant impact on both short- and long-term patient prognosis [29, 30]. Sjoding et al. pointed out that observational studies adjusting for illness severity and comorbidity closely approximated the true effect of the treatment under study [2]. A comorbidity assessment tool is easy to use and relies only on the patient’s history rather than complicated tests and examinations. The two most commonly used comorbidity assessment tools are the CCI score and Elixhauser [31, 32]. The CCI score is easily administered and has flexibility, resulting in wider utilization than other risk assessment tools. The CCI score’s original intent was to assess the mortality risk, and it can also facilitate the prioritization of care-management resources based on patient risk [8].

Compared with previous studies, our results conflict with some studies and are consistent with others. Currently, two large retrospective studies have shown that an increased CCI is associated with a decreased survival rate for OHCA [19, 33]. Emily Andrew et al.’s study, involving 15,953 nontraumatic OHCA patients, suggested that an increased CCI score was independently associated with an increased odds of in-hospital and discharge mortality and reduced 1-year functional recovery. The authors concluded that the consideration of comorbidities could improve the prediction of outcomes in patients with OHCA as well as other traditional predictors [19]. In the study of Oving et al. with 2,510 patients included, the higher CCI-score is related to the lower survival rate in the in-hospital phase, but is little related to the survival rate in pre-hospital phase [34]. The aforementioned studies all focused on patients with OHCA, while we in this article concentrated on the patients with IHCA instead. Eva Piscator et al. also suggested that the severity of the age-adjusted CCI score was correlated with a poor prognosis for in-hospital cardiac arrest [14]. However, Winther-Jensen M et al. found no correlation between the CCI score and the prognosis of cardiac arrest [18]. That study is a retrospective study of the target temperature management trial with 939 patients included, which is different from the patient population included in our study. The sample size of this study is smaller, which may be the reason for the varied research results. Idrees Salam’s study concluded that the CCI score only affects survival in patients with a primary non-shockable rhythm but has no significant impact on patients with a shockable rhythm [35]. While we in our study did not classify between different types of shockable rhythms, which may partially explain why we drew different results. Lars W. Andersen et al.’s review pointed out that the prognosis of patients with cardiac arrest is related to many factors, such as the patient population, withdrawal of care, and treatment during and after cardiac arrest [20]. According to previous studies, patients with fewer comorbidities were more likely to receive corresponding treatment, which may further affect the patients’ survival rate [18]. Consequently, the reasons for these inconsistent study results may be caused by the different research methods chosen by the authors of these studies, the methods used to calculate and propose the CCI score, the study population selection and the sample size.

Apart from focusing on the CCI score, we also found that the diagnosis of cardiac arrest due to underlying cardiac condition based on ICD-10 (with ICD-code equal to I462) was significantly associated with a good clinical prognosis, which is consistent with the analysis of the secondary indices of CCI scores reported in Table 1. The number of patients with myocardial infarct or congestive heart failure in the in-hospital survival group was higher than the number of corresponding patients in the in-hospital death group. This may be because more timely and effective treatment can be obtained in the hospital after the causes are determined. Generally, cardiac causes account for more than half of cardiac arrest occurrences, with common causes including myocardial infarction, arrhythmia and heart failure [20]. Recognizing a potential cardiac cause could improve patient outcomes [36]. Previous studies suggested that patients with in-hospital cardiac arrest caused by cardiovascular diagnoses had a better prognosis [37, 38]. Due to the inaccurate diagnoses of cardiac arrest based on the present ICD code, a more sophisticated diagnosis code to distinguish among different cardiac causes is needed.

Additionally, our study results showed that vital signs and the minimum level of lactate during ICU admission might be independent factors associated with a higher risk level of in-hospital mortality among patients with cardiac arrest. Existing studies have also suggested that early abnormal vital signs and admission lactate levels may be effective predictors of the outcome of patients experiencing cardiac arrest [25, 39–41]. Thus, continuous monitoring, early recognition of deterioration, and appropriate treatment to intervene with vital signs and lactate may be of great importance to improve the prognosis.

Our study also found that patients with chronic liver disease, malignant cancer or metastatic solid tumors were associated with poor outcomes, which is consistent with previous studies [19, 33, 38, 42]. Their deaths may be caused by a poor baseline health status or their primary diseases. Regarding other aspects, patients with a low BMI or who were undergoing mechanical ventilation also had a poor prognosis. A higher BMI may be a protective factor for critically ill patients in the ICU [43, 44], possibly because critically ill patients with a higher BMI can tolerate a lack of nutrition consumption. For airway management, a potential advantage of creating an advanced airway is that it helps maintain a continuation of chest compressions by reducing pauses [45], but the current evidence does not support early tracheal intubation in either in-hospital or out-of-hospital cardiac arrest [17, 46].

For the secondary outcomes, our study found that age-adjusted CCI was related to the days of stay in the hospital for patients with cardiac arrest [47, 48]. The cause of this phenomenon could be that patients with higher comorbidity indexes or more comorbidities need more complex treatments, which might require more time in the hospital. There are only limited studies focusing on patients with cardiac arrest, leaving plenty of questions to be answered in future studies. Other potential factors that may influence the length of stay in the hospital or ICU are the sepsis situation and the GCS score [49].

Finally, although our results showed that the age-adjusted CCI score may affect pathophysiology and the patients’ responses to treatment, we do not know whether and how comorbidities might alter treatment approaches to change the outcomes. Currently, there is no single index to estimate the prognosis of patients experiencing cardiac arrest, and other factors should be considered to form an effective prediction system. This study is based on unitary database and explore the relationship between age-adjust CCI and outcomes for ICU patients with cardiac arrest, and the purpose of the study is explorative which want to offer enlightenment for further tests based on larger sample [50]. As a result, we did not finish the test based on TRIPOD statement. And we have made the explanation in the article as well. Prospective studies that include comorbidity risk factors to assess the prognosis of patients with cardiac arrest are still needed in the future.

### Limitation

First, this study is a retrospective observational study with multiple potential biases, and there may be unmeasured confounders when evaluating the relationship between CCI and in-hospital death. Second, although our results showed that age-adjusted CCI was associated with in-hospital death in ICU patients with cardiac arrest, we could not identify the location or details of the initial treatment when cardiac arrests occurred. Meanwhile, the discharge diagnosis of the patient but not the admission diagnosis was used to calculate the age-adjusted CCI in our study, which cannot fully represent the basic comorbidity status of the patients. Thus, the current results may not demonstrate a causal relationship between comorbidity conditions and in-hospital mortality outcomes. Third, the research results came from a specific database and may not be generalizable. According to the present data obtained from the MIMIC-IV database, we cannot separate the in-hospital cardiac arrest (IHCA) and out-of-hospital cardiac arrest (OHCA). Finally, our study did not investigate the association between comorbidities and the long-term prognosis in cardiac arrest patients.

## Conclusion

In ICU patients with cardiac arrest, the age-adjusted CCI score was associated with in-hospital death and length of hospitalization stay, and it may be a valid indicator to predict mortality for those patients with cardiac arrest. Future studies are required to investigate how comorbidity status affects cardiac arrest outcomes.

## Data Availability

This study is a retrospective study based on MIMIC-IV database (https://physionet.org/content/mimiciv/1.0/), which has received ethical approval from the Institutional Review Boards (IRBs) at Beth Israel Deaconess Medical Center (BIDMC) and Massachusetts Institute of Technology (MIT). And individual patient consent was not needed because the database does not contain protected health information. The authors obtained permission to use the data.

## Abbreviations

CCI: Charlson comorbidity index
ICD: International Classification of Diseases
ICU: Intensive Care Unit
BMI: Body Mass Index
SBP: Systolic Blood Pressure
DBP: Diastolic Blood Pressure
HR: Heart Rate
RR: Respiratory Rate
T: Temperature
SpO2: Pulse Oxygen Saturation
GCS: Glasgow Coma Scale
Lac: Lactate
AIDS: Acquired Immune Deficiency Syndrome
MV: Mechanical Ventilation
OR: Odds Ratio
CI: Confident interval
VF: Ventricular fibrillation
VT: Ventricular tachycardia
IHCA: In-hospital cardiac arrest
OHCA: Out-of-hospital cardiac arrest
